# Quadri-Pulse Theta Burst Stimulation using Ultra-High Frequency Bursts – A New Protocol to Induce Changes in Cortico-Spinal Excitability in Human Motor Cortex

**DOI:** 10.1371/journal.pone.0168410

**Published:** 2016-12-15

**Authors:** Nikolai H. Jung, Bernhard Gleich, Norbert Gattinger, Catrina Hoess, Carolin Haug, Hartwig R. Siebner, Volker Mall

**Affiliations:** 1 Department of Pediatrics, Technical University of Munich, Heiglhofstr. 63, Munich, Germany; 2 Zentralinstitut für Medizintechnik at Technical University of Munich (IMETUM), Boltzmannstr. 11, Garching, Germany; 3 Danish Research Center for Magnetic Resonance, Copenhagen University Hospital Hvidovre, Kettegaard Allé 30, Hvidovre, Denmark; 4 Department of Neurology, Copenhagen University Hospital Bispebjerg, Copenhagen, Denmark; University of Ottawa, CANADA

## Abstract

Patterned transcranial magnetic stimulation (TMS) such as theta burst stimulation (TBS) or quadri-pulse stimulation (QPS) can induce changes in cortico-spinal excitability, commonly referred to as long-term potentiation (LTP)-like and long-term depression (LTD)-like effects in human motor cortex (M1). Here, we aimed to test the plasticity-inducing capabilities of a novel protocol that merged TBS and QPS. 360 bursts of quadri-pulse TBS (qTBS) were continuously given to M1 at 90% of active motor threshold (1440 full-sine pulses). In a first experiment, stimulation frequency of each burst was set to 666 Hz to mimic the rhythmicity of the descending cortico-spinal volleys that are elicited by TMS (i.e., I-wave periodicity). In a second experiment, burst frequency was set to 200 Hz to maximize postsynaptic Ca^2+^ influx using a temporal pattern unrelated to I-wave periodicity. The second phase of sinusoidal TMS pulses elicited either a posterior-anterior (PA) or anterior-posterior (AP) directed current in M1. Motor evoked potentials (MEPs) were recorded before and after qTBS to probe changes in cortico-spinal excitability. PA-qTBS at 666 Hz caused a decrease in PA-MEP amplitudes, whereas AP-qTBS at 666 Hz induced an increase in mean AP-MEP amplitudes. At a burst frequency of 200 Hz, PA-qTBS and AP-qTBS produced an increase in cortico-spinal excitability outlasting for at least 60 minutes in PA- and AP-MEP amplitudes, respectively. Continuous qTBS at 666 Hz or 200 Hz can induce lasting changes in cortico-spinal excitability. Induced current direction in the brain appears to be relevant when qTBS targets I-wave periodicity, corroborating that high-fidelity spike timing mechanisms are critical for inducing bi-directional plasticity in human M1.

## Introduction

Long-term changes in synaptic efficacy, commonly referred to long-term potentiation (LTP) and long-term depression (LTD) [1], are important neurophysiological substrates of learning and memory [2–4]. These changes (i.e. increase or decrease in cortico-spinal excitability) can be induced non-invasively in the human cortico-spinal motor system using continuous or patterned repetitive transcranial magnetic stimulation (rTMS) protocols displayed by an increase or decrease in MEP amplitudes [5, 6]. Two patterned rTMS protocols have been established to induce synaptic or cortical plasticity in human primary motor cortex (M1). Theta-burst stimulation (TBS) applies 50 Hz bursts consisting of three TMS pulses at a burst repetition rate of 5 Hz [7, 8]. Burst stimulation given at a repetition rate within the theta band (i.e., theta bursts) is also an effective stimulation protocol for induction of synaptic plasticity in animal models [8].

The other patterned stimulation protocol is called quadri-pulse stimulation (QPS), and consists of four-pulse bursts with interstimulus intervals (ISI) of 1.5–125 ms separated by long inter-burst intervals of several seconds [9]. Depending on the ISI, QPS was shown to produce an increase in cortical excitability at short ISI of 1.5, 5 or 10 ms and a decrease in cortico-spinal excitability at ISI of 30, 50, 100, or 125 ms. In addition to the burst repetition rate, TBS and QPS protocols also differ with respect to the pulse configuration of the TMS pulse. TBS is usually applied with a repetitive TMS stimulator which generates full-sine wave forms [7], while QPS uses monophasic wave forms [9].

Until recently, technical limitations prevented the application of QPS at shorter inter-burst intervals, for instance in the theta range. In this study, we took advantage of a new TMS device that enables the combination of TBS and QPS. Here, we tested novel quadri-pulse theta burst stimulation (qTBS) in healthy volunteers. We applied quadruple bursts at a burst-repetition rate of 5 Hz to the hand area of the primary motor cortex (M1-HAND) and probed the plasticity-inducing capabilities of qTBS with single-pulse TMS before and after qTBS. Quadri-pulses were given either at 666 Hz or 200 Hz corresponding to the two shortest ISIs (1.5 and 5.0 ms) that had been used for classic QPS to induce an increase in cortical excitability in M1 [9],

We hypothesized that qTBS at 666 Hz (corresponding to an ISI of 1.5 ms) would be optimal to induce plasticity in M1-HAND because a quadruple burst at an ISI of 1.5 ms mimics the rhythmic pattern of multiple descending volleys (so-called I-wave rhythmicity) that can be recorded in the cortico-spinal tract in response to single-pulse TMS [10, 11]. In other words, quadruple pulses at 666 Hz have the optimal rhythmicity to transsynaptically excite and hereby maximize postsynaptic Ca^2+^ influx into the cortico-spinal output neurons [12].

The threshold for inducing synaptic plasticity as well as the sign of synaptic plasticity (increase or decrease in cortico-spinal excitability) critically depends on the magnitude and temporal dynamics of the postsynaptic Ca^2+^ influx induced by presynaptic stimulation [13]. Tetanization frequencies of 200 Hz demonstrated to lead to Ca^2+^ dependent forms of synaptic plasticity [8]. Therefore, we decided to apply qTBS using 200 Hz bursts (corresponding to an ISI of 5ms) to optimize postsynaptic Ca^2+^ influx as a precondition of an increase in synaptic strength (i.e. LTP formation) [14, 15], but without introducing a temporal pattern of excitation that is reminiscent of intrinsic I-wave periodicity.

We further hypothesized that the preferentially induced current direction in M1-HAND may be another important variable that determines the efficacy of qTBS to induce changes in cortico-spinal excitability in human M1. We reasoned that this may apply particularly to qTBS at 666 Hz, targeting I-wave periodicity. A single TMS pulse targeting the M1-HAND elicits multiple descending volleys in the cortico-spinal tract [11]. The recruitment pattern of these multiple descending volleys critically depends on the current direction induced in M1-HAND [10]. A TMS pulse that preferentially induces a posterior-anterior (PA) current in M1-HAND primarily leads to a transsynaptic recruitment of an early I-wave (I1). If the induced current has an anterior-posterior (AP) direction, later I-waves (I3) are primarily recruited [16, 17]. Recently, it has been demonstrated that TMS recruiting preferentially I1 or I3 waves, pre-determines the ability of an interventional rTMS protocol to induce an in- or decrease in cortico-spinal excitability in human primary motor cortex [12, 18, 19]. I3 waves are considered to be primarily involved in an increase in cortico-spinal excitability whereas I1 waves are considered to be involved in a decrease in cortico-spinal excitability [18]. We hypothesized that flipping the current direction in the brain will lead to different effects in cortico-spinal excitability in human M1.

## Material and Methods

### Participants

**Fig 1:**
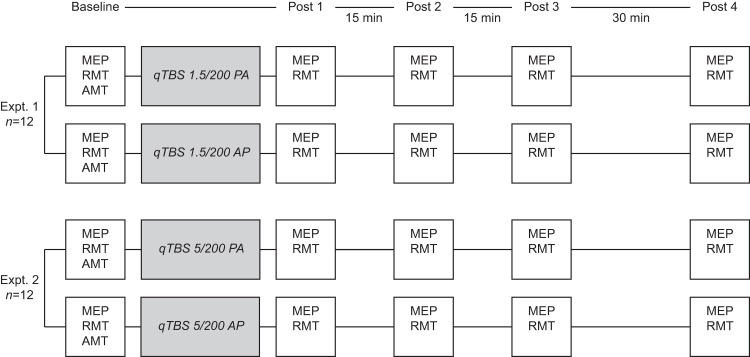
Experimental procedures, timeline of experiments and number of participants for each experiment. We performed two sets of experiments. In experiment 1, the interstimulus interval (ISI) of each pulse was set to 1.5 ms (666 Hz) to test I-wave frequency dependent patterns of qTBS. In experiment 2, the ISI was set to 5 ms and consequently suggested to be outside I-wave periodicity. In all experiments, the current flow in the brain was reversed from PA to AP. MEP: motor evoked potential, RMT: resting motor threshold, AMT: active motor threshold.

Sixteen healthy volunteers (11 women, 5 men) aged 18 to 36 years (median 23.5 years [SD 3.9]) participated in the study. Twelve volunteers participated in each experiment (i.e. qTBS with 1.5 ms or 5 ms ISI in AP and PA direction, respectively) with eight participants taking part in all experiments (Fig 1). All participants gave written informed consent before their participation. The study was approved by the local Ethics Committee of the Technical University of Munich, Faculty of Medicine (vote 5423/12) and carried out according to the latest version of the Declaration of Helsinki. All participants except one were right-handed according to the Edinburgh Handedness Inventory [20]. A structured interview conformable to existing guidelines revealed neither a history of neurological or psychiatric illnesses nor any exclusion criteria concerning the safety of TMS [21].

### Experimental procedures

We conducted two experiments in the present study to investigate frequency dependency of qTBS and to find out whether it demonstrates I-wave dependent bi-directionality in human M1. For detailed timelines see Fig 1. In experiment 1, the interstimulus interval (ISI) of each pulse was set to 1.5 ms (666 Hz) to match intrinsic I-wave periodicity [11]. In experiment 2, the ISI was set to 5 ms and consequently suggested to be outside I-wave periodicity, but optimal for producing a strong postsynaptic Ca^2+^ influx. The order of sessions with different stimulation paradigms was randomized and counterbalanced between subjects with a minimum intersession period of one week to avoid carry-over effects. Participants were blinded to the experimental condition. We recorded MEP amplitudes and resting motor threshold before (pre-interventional baseline) as well as at four time points after the end of qTBS (post-qTBS) in PA and AP direction (post 1, 0 min; post 2, 15 min; post 3, 30 min; post 4, 60 min). Current direction in the brain (i.e. AP and PA, respectively) was always the same for evaluation and intervention. There was no change between evaluation and intervention (e.g. AP current flow for evaluation and PA current flow for qTBS and vice versa).

### Electromyographic recording

Participants were seated comfortably in a chair reposing both hands suitably on a cushion or their lap to ensure complete relaxation. Motor evoked potentials (MEP) were recorded by surface electromyography (EMG) from the non-dominant abductor pollicis brevis (APB) muscle using silver/silver chloride surface electrodes (surface area 263 mm^2^; AMBU, Ballerup, Denmark) mounted in belly-tendon technique. Participants were asked to relax the target muscle during measurement. Data were bandpass filtered (20–2000 Hz) and amplified using an Ekida DC universal amplifier (Ekida, Helmstadt, Germany) connected to a Micro 1401 *mk*II data acquisition unit (Cambridge Electronic Design, Cambridge, UK) with a sampling rate of 5 kHz and stored on a personal computer for online visual display and later offline analysis using Signal software version 5 (Cambridge Electronic Design). MEP size was determined by measuring the two highest peaks of opposite polarity [22] and then averaged over 20 trials. Trials that differed by over three times the standard deviation from the mean were considered as outliers and were excluded from analysis [23].

### Transcranial magnetic stimulation

For focal TMS the intersection of a figure eight shaped stimulation coil with an outer diameter of 100 mm was centered tangentially on the scalp over the primary motor cortex of the non-dominant hand (M1-HAND) with its handle pointing in a posterior direction and laterally at an angle of approximately 45° away from the midline. Thus, the current in the brain induced by TMS flowed roughly perpendicular to the presumed line of the central sulcus.

**Fig 2:**
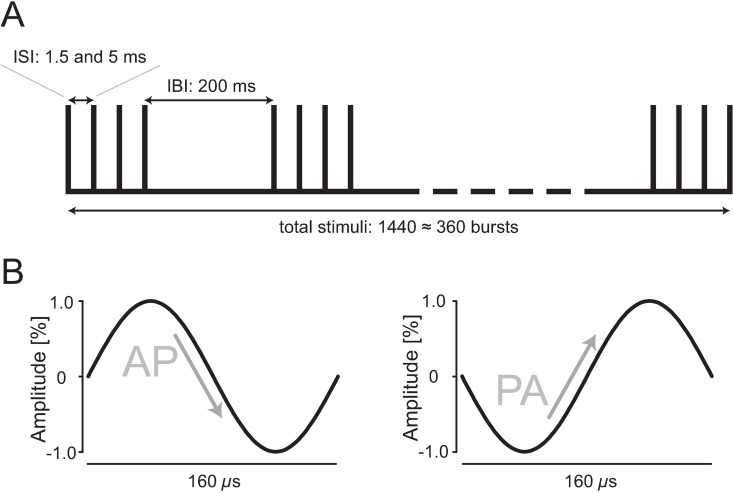
Schematic drawing of qTBS pulse sequence and of current waveforms. (A) The conditioning protocol of qTBS comprises 360 trains of TMS pulses. Each train consists of four biphasic magnetic pulses (i.e. quadri-pulse stimulation; QPS) delivered at interstimulus intervals of 1.5 ms and 5 ms resulting in a total of 1440 stimuli. Trains were repeated every 200 ms. (B) Each stimulus had a biphasic current waveform with 160 μs pulse duration. To reverse current direction from PA to AP and vice versa, a cable was connected to the coil changing the polarity of the induced current, hereby flipping the direction of the induced current in the M1-HAND. We refer to PA stimulation when the initially induced current in M1-HAND had a posterior-to-anterior direction and, conversely, AP stimulation refers to stimulation producing an anterior-to-posterior current flow in M1-HAND. For clarity, current direction always refers to the direction of current induced in M1-HAND by the second component of the induced current covering the second and third quarter of the biphasic cycle. ISI: interstimulus interval, IBI: interburst interval.

The coil was connected to a custom made magnetic stimulation device (QuattroMag, Zentralinstitut für Medizintechnik at Technical University of Munich, IMETUM, Munich, Germany) with a biphasic current waveform with 160 μs pulse duration (Fig 2). The device was used for single pulse TMS evaluation as well as for repetitive qTBS stimulation. To reverse current direction from PA to AP, a cable was connected to the coil changing the polarity of the induced current, hereby flipping the direction of the induced current in the M1-HAND. We refer to PA stimulation when the initially induced current in M1-HAND had a posterior-to-anterior direction and, conversely, AP stimulation refers to stimulation producing an anterior-to-posterior current flow in M1-HAND (Fig 2). For clarity, current direction always refers to the direction of current induced in M1-HAND by the second component of the induced current covering the second and third quarter of the biphasic cycle because it appears to be most relevant due to its long duration [17].

At the beginning of each experiment, we determined the optimal site for TMS of the M1-HAND by administering slightly suprathreshold single-pulse stimuli. We gradually moved the coil over the presumed M1-HAND representation until we located the position where a single TMS pulse elicited MEP of maximum amplitudes in the target muscle. This position of the coil was marked on the scalp with a felt-tip pen. Magnetic stimuli for evaluation were administered to the cortex at a frequency of 0.1 Hz with a variation of 15% to avoid habituation. Stimuli used to identify hotspot and motor thresholds were administered at 0.25 Hz. We then determined the resting motor threshold (rMT) using a maximum-likelihood threshold-hunting procedure [24] with the TMS Motor Threshold Assessment Tool (MTAT 2.0) available from (Awiszus F, Borckardt JJ. TMS Motor Threshold Assessment Tool (MTAT 2.0). Available at: http://www.clinicalresearcher.org/software.htm; 2006 [accessed on: 10-20-2011].). Sixteen TMS stimuli were applied starting at 45% of maximum stimulator output (MSO). A MEP was defined as a potential larger than 50 μV in peak-to-peak amplitude. Active motor threshold (AMT) was then determined. It was defined as the lowest intensity that evoked a small response (>100 μV) when the subjects maintained a slight contraction of the APB (5–10% of the maximum voluntary contraction) [9], as controlled by a manometer and observed by means of a monitor, using the same threshold-hunting procedure. Following determination of motor threshold, the stimulator output for evaluation was then adjusted to elicit mean MEP amplitudes of 800–1200 μV peak-to-peak (SI_1mV_).

### Quadri-pulse TBS (qTBS) and experimental design

QTBS was applied with a biphasic waveform over M1-HAND. The patterns of qTBS consisted of bursts containing four pulses at the same intensity separated by 1.5 ms (~666 Hz) and 5 ms (200 Hz), respectively. Each burst was separated by 200 ms (5 Hz) (Fig 2). The stimulus intensity of each pulse was set to 90% AMT as previously described for quadri-pulse stimulation [9]. A total of 1440 pulses were delivered in each session, resulting in 360 bursts. To probe the direction effect of qTBS, we changed the effective direction flow in the brain of the biphasic pulse from PA to AP as mentioned above.

### Analyses and statistics

Relaxation of the target muscle was monitored online using visual feedback of the electromyographic activity recorded from the APB. Additionally, each MEP sweep was inspected offline for the presence of voluntary muscle activity. If the MEP sweep showed electromyographic activity exceeding 0.05 mV, the trial was excluded from further analyses. In total n = 119 trials in 48 qTBS sessions with 5 measuring timepoints (pre and post 1–4) were excluded from further analyses. The pre-stimulus time window for determining if MEP were contaminated by muscle activity was 120 ms.

We computed all statistical analyses using IBM SPSS Statistics software, version 20.0 (IBM SPSS statistics Inc., Chicago, IL, USA). Statistical evaluation of qTBS data was performed using repeated-measure analysis of variance (ANOVA) with the inner-subject factors TIME (5 levels: PRE, POST 1, POST 2, POST 3, POST 4) and DIRECTION (2 levels: PA and AP) after the Kolmogorov-Smirnov test revealed no violations of the assumption of normality. No transformations were required. All statistics were performed with the mean MEP amplitude of each case consisting of 20 MEP trials or of rMT values (%MSO) averaged to a mean. Accordingly, figures display the mean MEP amplitude or rMT of all cases. If necessary, we used the Greenhouse-Geisser correction to adjust for violations of sphericity, resulting in adjusted *p*-values based on adjusted degrees of freedom. In case of significant main effects or interactions, we conducted *post hoc* two-tailed paired *t*-tests for PRE-POST investigations, and *post hoc* two-tailed unpaired *t*-tests for inter-group comparisons with Bonferroni correction for multiple comparisons. This method was used for MEP and resting motor threshold data. Significance level was set at *α* = 0.05. All values given are mean group values ± SD, if not indicated otherwise.

## Results

No participant of the present study reported any adverse events during or after the experiments. Number, pattern and details of each experiment are illustrated in Fig 1. Mean baseline data of MEP did not differ significantly among experiments prior to the intervention (for details see mean MEP values and standard deviations below). Individual data of each participant are presented as figures in a supplementary information file of this article. No changes in hotspots between AP and PA direction were observed. Mean TMS intensities (%MSO) for each condition (SI1mV) are depicted below.

### QTBS at ISI 1.5 ms in PA and AP direction

**Fig 3:**
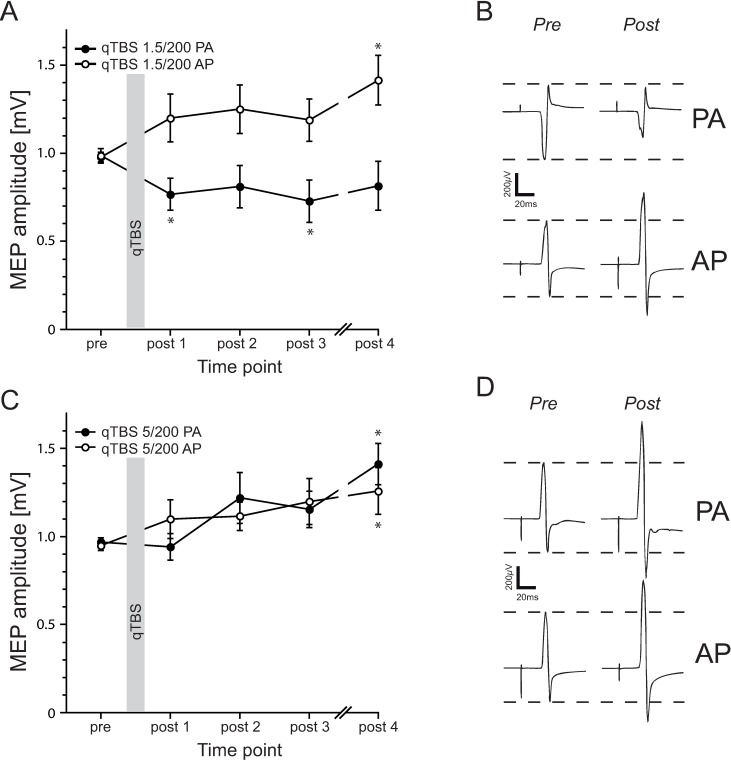
Results of MEP data after qTBS. (A) Timecourse of MEP amplitudes after qTBS at ISI of 1.5 ms in AP and PA direction. Flipping the current flow in M1-HAND led to bi-directional changes in synaptic plasticity with a significant increase of cortico-spinal excitability in AP direction and a significant decrease in PA direction. (B) MEP amplitudes of one representative subject before and after (POST4) qTBS 1.5 ms in AP and PA direction. Shown are averages of 20 MEP trials in each case. (C) Timecourse of MEP amplitudes after qTBS at ISI of 5 ms in AP and PA direction. In both current directions (i.e. AP and PA), MEP amplitudes significantly increased after qTBS. (D) MEP amplitudes of one representative subject before and after (POST4) qTBS at ISI of 5 ms in AP and PA direction. Shown are mean amplitudes of averages of 20 MEP trials in each case. Asterisks indicate significant differences between pre and post measurements (*p*<0.05, paired *t*-test). Error bars indicate the standard error of the mean (S.E.M.). qTBS: quadri-pulse theta burst stimulation; PA: posterior-anterior; AP: anterior-posterior; ISI: interstimulus interval; MEP: motor evoked potential.

**Fig 4:**
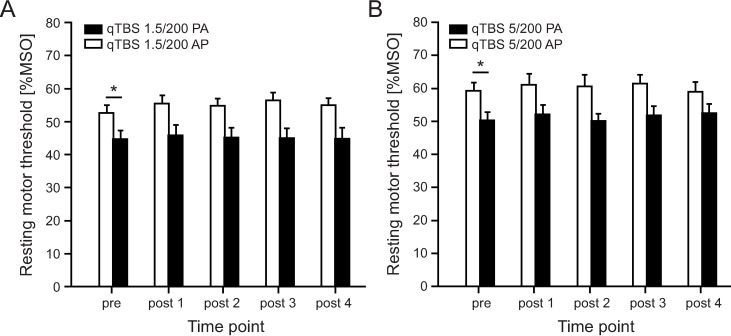
**Motor threshold following qTBS in AP and PA direction in qTBS at 1.5 ms (A) and 5 ms (B) interstimulus intervals.** No significant changes in rMT were observed after qTBS. MT in AP and PA direction significantly differed at pre measurements as expected. qTBS: quadri-pulse theta burst stimulation; PA: posterior-anterior; AP: anterior-posterior.

Twelve volunteers participated in this experiment to assess the effect of bi-directionality of qTBS at I-wave latencies (i.e. at ISI 1.5 ms). Mean TMS intensity was 79.9 %MSO ± 11.9 for SI1mV in AP direction and 69 %MSO ± 15.6 for SI1mV in PA direction. RmANOVA of MEPs showed a significant main effect of DIRECTION (*F*_1;11_ = 12.791, *p* = 0.004) and TIME x DIRECTION interaction (*F*_4;44_ = 4.859, *p* = 0.002) but not for TIME (*F*_4;44_ = 1.017, *p* = 0.409). MEP amplitudes significantly increased (mean MEP amplitude in AP direction: pre: 0.98 mV ± 0.10, post 1: 1.20 mV ± 0.47, post 2: 1.25 mV ± 0.48, post 3: 1.19 mV ± 0.42, post 4: 1.41 mV ± 0.49) on time point POST 4 (post hoc *t*-test: *p* = 0.014) in AP direction and significantly decreased (mean MEP amplitude in PA direction: pre: 0.99 mV ± 0.14, post 1: 0.77 mV ± 0.32, post 2: 0.81 mV ± 0.42, post 3: 0.73 mV ± 0.42, post 4: 0.81 mV ± 0.49) on time points POST 1 and POST 3 (post hoc *t*-test: *p* = 0.008, *p* = 0.037, respectively) in PA direction (Fig 3A and 3B). RmANOVA on rMT data revealed a significant main effect of DIRECTION (*F*_1;11_ = 7.203, *p* = 0.021) but not for TIME (*F*_4;44_ = 0.973, *p* = 0.432) and TIME x DIRECTION interaction (*F*_4;44_ = 4.528, *p* = 0.210). As expected, baseline data of rMT prior to qTBS differed significantly (post hoc *t*-test: *p* = 0.012), with a higher threshold in AP direction (Fig 4A). Mean rMT in AP direction: pre: 59.33 %MSO ± 8.40, post 1: 61.00 %MSO ± 11.98, post 2: 60.58 %MSO ± 12.19, post 3: 61.33 %MSO ± 9.74, post 4: 59.00 %MSO ± 10,04. Mean rMT in PA direction: pre: 50.25 %MSO ± 8.76, post 1: 52.17 %MSO ± 9.63, post 2: 50.17 %MSO ± 7.35, post 3: 51.83 %MSO ± 9.75, post 4: 52.42 %MSO ± 10.17.

### QTBS at ISI 5 ms in PA and AP direction

In this experiment, we investigated the effect of the current flow direction on qTBS at ISI of 5 ms (i.e. pulse repetition rate of 200 Hz) which is considered to be outside the range of I1-3 periodicity but is expected to trigger maximal postsynaptic Ca^2+^ influx during a single burst. Mean TMS intensity was 73.4 %MSO ± 15.9 for SI1mV in AP direction and 63.6 %MSO ± 12.3 for SI1mV in PA direction. In 12 volunteers, a significant increase in MEP amplitudes after stimulation on time point POST 4 in PA direction (post hoc *t*-test: *p* = 0.003) and AP direction (post hoc *t*-test: *p* = 0.049) was observed (mean MEP amplitude in AP direction: pre: 0.95 mV ± 0.10, post 1: 1.10 mV ± 0.38, post 2: 1.12 mV ± 0.28, post 3: 1.20 mV ± 0.45, post 4: 1.26 mV ± 0.46; mean MEP amplitude in PA direction: pre: 0.97 mV ± 0.08, post 1: 0.94 mV ± 0.26, post 2: 1.22 mV ± 0.50, post 3: 1.15 mV ± 0.36, post 4: 1.41 mV ± 0.41), with rmANOVA showing a significant main effect of TIME (*F*_4;44_ = 4.652, *p* = 0.03) and not for DIRECTION (*F*_1;11_ = 0.028, *p* = 0.871) and TIME x DIRECTION interaction (*F*_4;44_ = 1.382, *p* = 0.256) (Fig 3C and 3D).

RMT did not change after stimulation in both current directions (rmANOVA: TIME (*F*_4;44_ = 2.350, *p* = 0.069), DIRECTION (*F*_1;11_ = 12.413, *p* = 0.005) and TIME x DIRECTION interaction (*F*_4;44_ = 1.264, *p* = 0.298) but differed prior to stimulation with expected higher values in AP direction (post hoc *t*-test, PRE: *p* = 0.008 (Fig 4B). Mean rMT in AP direction: pre: 52.58 %MSO ± 8.28, post 1: 55.50 %MSO ± 8.32, post 2: 54.75 %MSO ± 7.64, post 3: 56.50 %MSO ± 8.10, post 4: 55.00 %MSO ± 7.52. Mean rMT in PA direction: pre: 44.67 %MSO ± 9.00, post 1: 45.75 %MSO ± 10.96, post 2: 45.08 %MSO ± 10.46, post 3: 45.00 %MSO ± 10.44, post 4: 44.75 %MSO ± 11.68.

## Discussion

Continuous qTBS consisting of ultra-high frequency bursts with a stimulus repetition rate of 666 Hz or 200 Hz can induce lasting changes in cortico-spinal excitability in human M1. The induced current direction in the brain determined the direction of the plasticity response when stimulus repetition rate matched I-wave periodicity. AP-qTBS with 666 Hz bursts caused an increase in cortico-spinal excitability, whereas PA-qTBS at 666 Hz induced a decrease in cortico-spinal excitability. At a lower stimulus repetition rate of 200 Hz, qTBS also was effective but the plasticity-inducing effects were independent of the current direction. Both, AP-qTBS and PA-qTBS with 200 Hz bursts produced an increase in cortico-spinal excitability displayed by an increase in MEP amplitudes.

### Bi-directional changes following I-wave specific qTBS with 666 Hz

Recent research provided evidence that the preferential recruitment (i.e. of I-waves I1 or I3 waves) pre-determines the ability of an interventional rTMS protocol to induce an increase or decrease in cortico-spinal excitability in human primary motor cortex [12, 18, 19, 25].

Monophasic QPS and biphasic TBS have previously demonstrated to induce lasting alterations in cortico-spinal excitability, referring to a model of LTP- or LTD- like changes [7, 9]. A single TMS pulse can preferentially target early or late I-waves (i.e. I1 and I3 waves) by changing the direction of the current flow in the brain from posterior-anterior (PA) to anterior-posterior (AP) [10, 18, 26, 27]. This approach is well established and shows stable effects for monophasic, half-sine and biphasic pulse waveforms [16–18, 26].

Our study extends previous work in this field by applying a novel qTBS protocol that produces quadruple bursts matching the intrinsic frequency of I-waves, namely 666 Hz. We observed a bi-directionality of the plasticity-inducing effects of continuous qTBS at I-wave periodicity (666 Hz) but not at a lower frequency (200 Hz). The preferential current direction in the brain produced by biphasic pulses determined the direction of cortico-spinal plasticity.

Mainly two mechanisms have been discovered to highlight the synaptic plasticity relation to I-waves. First, different interneuron networks seem to drive human synaptic plasticity and, second, high fidelity spike timing dependent mechanisms are crucial for successful plasticity induction by TMS at I-wave periodicity [18, 25].

Our findings of bi-directional changes in cortico-spinal excitability by reversing the current direction in the brain are well in line with recently discovered mechanisms of different sets of interneurons that are targeted by I1 and I3 activation, and with findings from paired pulse studies discovering subliminal I-wave activation as a contributor to SICF which is suggested to modulate synaptic changes in human M1 [28]. It has been shown that suppression of early I-waves follows cTBS and facilitation of late I-waves follows iTBS [29, 30].

The results support the hypothesis of I3 waves being primarily involved in an increase and I1 waves being primarily involved in a decrease in cortico-spinal excitability. When 666 Hz bursts mainly induced a PA current in M1, it has to be assumed that PA-qTBS preferentially stimulated neural structures involved in early I-wave generation and exerted a decrease in cortico-spinal excitability. In contrast, when 666 Hz bursts mainly induced AP currents in M1, AP-qTBS primarily excited late I-waves, producing an increase in cortico-spinal excitability. We hypothesize that burst stimulation at I-wave periodicity preferentially “resonated” in neural structures involved in I1-wave or I3-wave generation by summation of the excitatory effect during the train.

Another contributing mechanism may be the need for a tight temporal coupling between pre- and postsynaptic events during burst stimulation at I-wave periodicity (i.e. spike timing dependent mechanisms) [25]. The model of high-fidelity spike-timing dependent plasticity has been previously introduced to so-called I-wave specific (i)TMS with pairs of pulses with a deviation of 0.5 ms of the maximum I-wave peak led to bi-directional effects [25]. However, these mechanisms remain somewhat speculative and need to be confirmed by further studies.

### Induction of cortico-spinal plasticity by continuous qTBS below I-wave periodicity

When quadruple bursts had a lower repetition rate of 200 Hz, continuous qTBS of M1 consistently produced an increase in cortico-spinal excitability regardless of the preferentially induced current direction in M1. It has been proposed that the anisotropy of the human M1 accounts for a preferential responsiveness of human M1 to the plasticity-inducing effects of rTMS [17]. Anteriorly directed currents (PA) are suggested to be soma-depolarizing and, conversely, posteriorly directed currents (AP) in M1 are soma-hyperpolarizing [17].

A preponderant increase in cortico-spinal excitability may be attributed to the stimulus repetition rate, resulting in an inter-stimulus interval of 5 ms. This burst pattern is considered to be optimal for *N*-methyl-D-aspartate (NMDA)-receptor activation inducing maximal postsynaptic Ca^2+^ influx, the main intracellular signal for the induction of LTP [15]. At the same time, an interburst interval of 5 Hz secures a sufficiently long pause between bursts preventing a decay in burst efficiency for instance by intracortical GABAergic inhibition. The theta burst frequency has also shown to be optimal for information storage in the mammalian brain supported by findings of hippocampal slice preparations [15]. Hence, continuous 200 Hz qTBS may represent an optimally tuned protocol to increase cortico-spinal excitability and neutralizes the impact of current-specific effects in human M1.

### Differences to previous findings

Our new qTBS protocol merged the classic QPS introduced by Hamada and co-workers [9] and conventional TBS developed by Huang and co-workers [7]. The classic protocol of monophasic QPS [9] was limited to an interburst frequency of 0.2 Hz. Here we established qTBS allowing the combination of fast interstimulus intervals (1,5 ms or 5 ms) as well as fast interburst intervals (200 ms).

How to compare the after-effects that were induced by qTBS to previous work using standard quadruple pulse stimulation [9]? Only the qTBS protocol with an inter-stimulus interval of 5 ms resulted in consistent increases in cortico-spinal excitability mirroring the effects as demonstrated in monophasic QPS. One would have also expected consistent facilitatory effects for 666 Hz qTBS in analogy to monophasic QPS. This was, however, only the case when biphasic qTBS at 666 Hz preferentially induced an AP current direction in M1. When qTBS at 666 Hz produced a preferential PA current direction, biphasic qTBS triggered a decrease in cortico-spinal excitability. This discrepancy suggests that different rules govern the plasticity-inducing effects of monophasic QPS and biphasic qTBS at I-wave periodicity.

Our novel qTBS protocol differed from standard quadruple pulse stimulation [9] in several aspects. First, we use a much shorter interburst interval of 200 ms which we considered to be optimal according to animal and human studies [7, 31]. Second, we applied biphasic rather than monophasic pulses. We chose two short inter-stimulus intervals, namely 1.5 ms and 5 ms, resulting in 666 Hz and 200 Hz quadruple bursts. Given the multiple differences, we cannot assign the divergent plasticity-induced effects to a single feature of qTBS. The difference in stimulus waveforms may be the central point for the different findings. Monophasic pulses may be of higher selectivity with respect to early and late I-wave generation. Biphasic pulses elicit different sets of neurons by the first quarter of the pulse. Hence, we may have elicited not solely I1 waves in PA and I3 waves AP stimulation but all I-waves with a preference for I1 or I3 waves, respectively. The lower I-wave selectivity of biphasic pulses may contribute to high-fidelity order effects because early I-waves may fall in between phases of refractoriness and non-refractoriness of neurons connecting to the primary motor neuron.

### Limitations

Limitations of the current study are that we did not evaluate MEP of the opposite current direction as compared to the intervention (qTBS). Hence, we are unable to draw a conclusion of how a stimulation of I1 circuits by a PA current flow may affect I3 circuits, and vice versa. However, here we aimed to investigate current specificity of a newly developed non-invasive brain stimulation protocol. Further studies may address this point as well as replicate the results.

Previous studies found that responses to plasticity inducing protocols are variable. This has been demonstrated for TBS [18] as well as for QPS [32]. One might argue that this may also apply for qTBS. Here, we tried to minimize the possibility that effects are only an “occasional observation” by choosing an intra-subject design with the same participants being investigated in AP and PA direction in both experiments. Participants were not screened for their individual response (i.e. responders) and no hints point towards a dependence of the effects depending on the population sampled. We tried to control, for example, for circadian effects by measuring the participants, if possible, at the same time of the day. In addition, we measured MEP amplitudes of the abductor digiti minimi muscle (ADM) at the same time to control for topographic effects of qTBS stimulation and found no effects in distant muscles (data not shown). Even in small sample sizes, it has been demonstrated that clear and reproducible plasticity effects can be observed with physiological and neurobiological meaningful protocols [33, 34].

The present study, however, did not aim to demonstrate superiority of qTBS over other rTMS and non-invasive brain stimulation (NIBS) protocols and, from the existing data, we do not consider it as a more optimal alternative to either TBS or QPS without further investigation. Rather, we aimed to investigate the after effects on cortico-spinal excitability and their direction dependency in a new and physiologically driven TMS protocol.

## Conclusion

We introduce a novel qTBS protocol that uses ultra-high frequency bursts and can effectively induce changes in cortico-spinal excitability in human M1. The induced current direction in the brain determines the sign of qTBS-induced plasticity, when the repetition rate of qTBS-bursts targets I-wave periodicity (i.e. 666 Hz bursts), supporting the notion that high-fidelity spike timing mechanisms are critical to bi-directional plasticity in human M1. When the repetition rate of qTBS-bursts is adjusted to maximize burst-induced Ca^2+^ influx into the postsynaptic cells (i.e., 200 Hz bursts), qTBS induces a lasting increase in cortico-spinal excitability independently from the current flow in the brain.

## Supporting Information

S1 Fig Figures with individual timecourses of MEP amplitudes after quadri-pulse theta burst stimulation. (PDF)
