# Supplementary material for: Quadri-Pulse Theta Burst Stimulation using Ultra-High Frequency Bursts – A New Protocol to Induce Changes in Cortico-Spinal Excitability in Human Motor Cortex
Source: PLoS One. 2016 Dec 15;11(12):e0168410. doi: 10.1371/journal.pone.0168410 (PMC5158069; doi:10.1371/journal.pone.0168410)
Supplement: S1 Fig — (PDF) [file pone.0168410.s001.pdf]

Individual timecourses of MEP amplitudes after quadri-pulse theta burst stimulation in AP and PA direction for interstimulus intervals of 1.5 ms interburst intervals of 200 ms

1a)

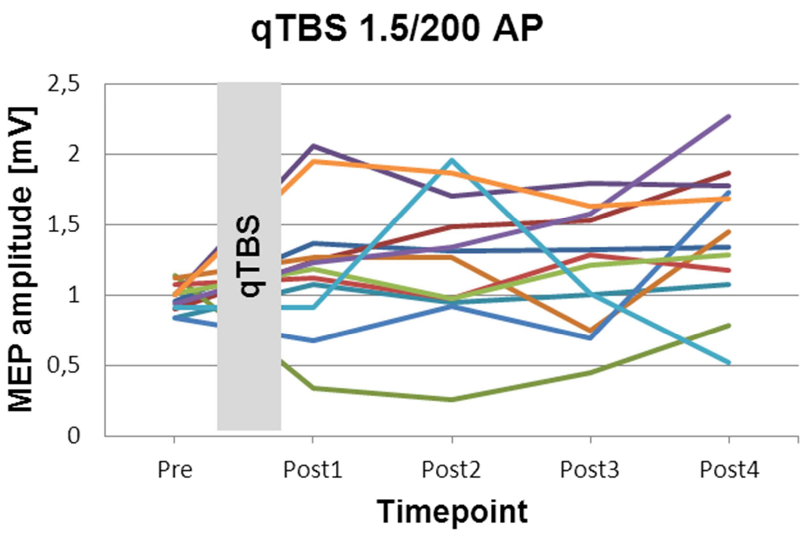

1b)

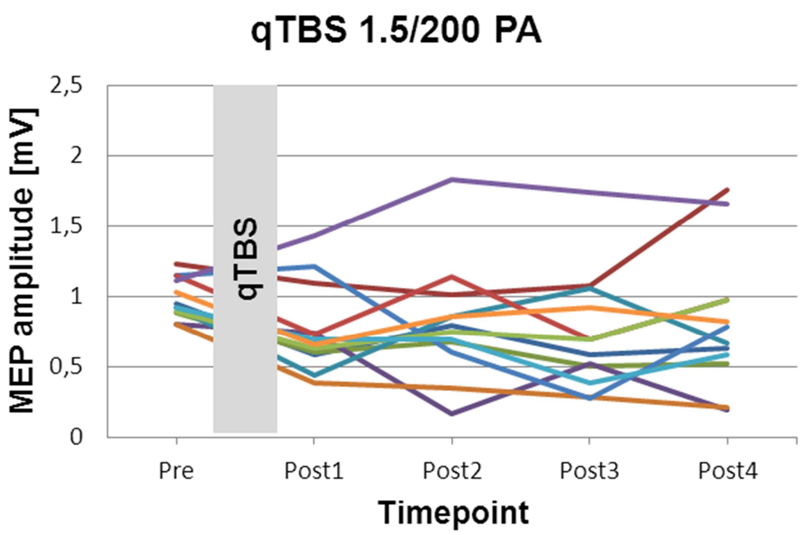

Figure 1a and b): Results of individual MEP data after qTBS with interstimulus interval of 1.5 ms in **a)** AP current direction and **b)** PA current direction in the brain. Each coloured line represents one participant. qTBS: quadri-pulse theta burst stimulation; AP: anterior-posterior; PA: posterior-anterior; MEP: motor evoked potential.

Individual timecourses of MEP amplitudes after quadri-pulse theta burst stimulation in AP and PA direction for interstimulus intervals of 5 ms and interburst intervals of 200 ms

2a)

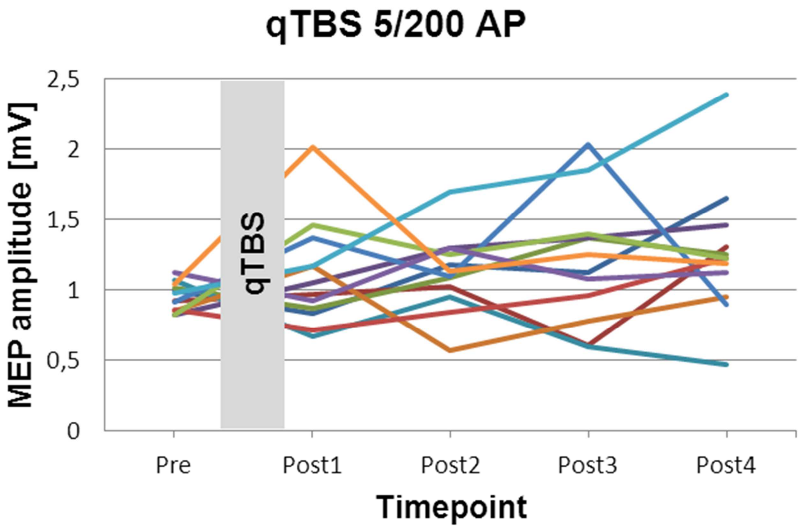

2b)

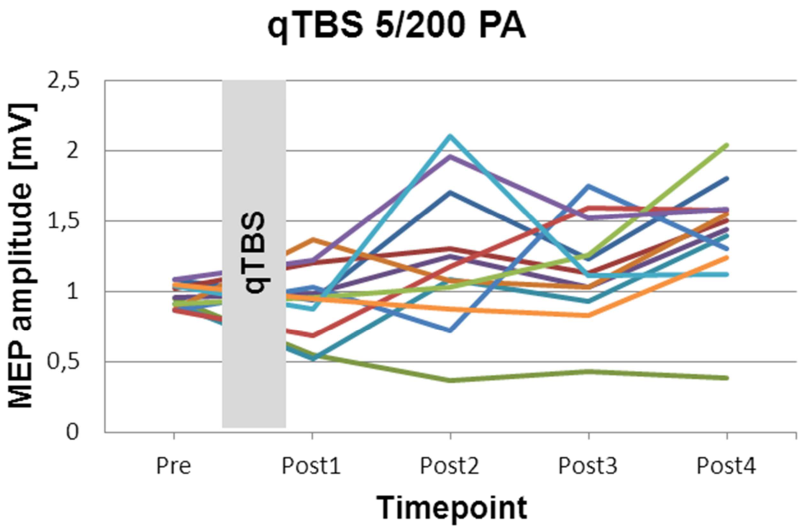

Figure 2a and b): Results of individual MEP data after qTBS with interstimulus interval of 5 ms in **a)** AP current direction and **b)** PA current direction in the brain. Each coloured line represents one participant. qTBS: quadri-pulse theta burst stimulation; AP: anterior-posterior; PA: posterior-anterior; MEP: motor evoked potential.
